# Longitudinal efficacy of risdiplam treatment in Chinese children with spinal muscular atrophy

**DOI:** 10.1186/s13023-025-03929-4

**Published:** 2025-07-31

**Authors:** Yue Yan, Danhui Zhu, Kai Ma, Xufeng Luo, Xiaoli Zhang, Xiaolong Deng, Mei Lu, Yang Li, Jianwei Li, Feng Gao, Shanshan Mao

**Affiliations:** 1 Department of Neurology, Children’s Hospital, Zhejiang University School of Medicine, National Clinical Research Center for Child Health, Hangzhou, Zhejiang 310052 China; 2 Department of Neurology, Children’s Hospital Affiliated to Shandong University, Jinan, Shandong 250022 China; 3 Department of Pediatric Neurology, Shenzhen Children’s Hospital of China Medical University, Shenzhen, 518000 China; 4 Department of Pediatric Neurology, The Third Affiliated Hospital of Zhengzhou University, Zhengzhou, Henan, 450052 China; 5 Department of Pediatric Neurology, Tongji Medical College, Wuhan Children’s Hospital, Huazhong University of Science and Technology, Wuhan, Hubei 430027 China; 6 Department of Pediatrics, Women and Children’s Hospital, School of Medicine, Xiamen University, Xiamen, Fujian 361000 China; 7 Department of Neurology, Children’s Hospital of Nanjing Medical University, Nanjing, Jiangsu 210000 China; 8 Department of Neurology, Dongguan Children’s Hospital, Xihu 3rd Road, Shilong Town, Dongguan, Guangdong 523320 China

**Keywords:** Risdiplam, Spinal muscular atrophy, Efficacy, Multicenter, FIREFISH

## Abstract

**Background:**

As the latest drug available for the treatment of spinal muscular atrophy (SMA), real-world research data on risdiplam are still lacking. The purpose of this study was to supplement the real-world data in SMA children receiving risdiplam by studying children in multiple centers throughout China.

**Results:**

In total, 34 children with SMA were collected from September 2021 to November 2024 and followed for a period of 8.3 ± 4.6 months. The Children’s Hospital of Philadelphia infant test of neuromuscular disorders (CHOPINTEND) scores of 18 children at the last visit improved when compared with baseline [20 (3–60) vs. 39 (8–61), *p* < 0.001]. Thirteen patients (76.5%) demonstrated a noticeable improvement in Hammersmith functional motor scale expanded (HFMSE) score at the last follow-up compared with baseline [22 (6–52) vs. 31 (8–59), *p* = 0.003]. Revised upper limb module (RULM) scores of 7 patients at the last follow-up were improved compared with those at baseline [21 (6–32) vs. 24 (9–35), *p* = 0.018]. Improvements in motor function were monthly quantified by generalized estimating equation analysis. The CHOPINTEND score increased by 1.8 points/month (95%CI 0.8 to 2.9, *p* = 0.001) in children with short disease duration before treatment, 2.7 points/month (95%CI 2.4 to 3.0, *p* < 0.001) in children with type 1 and 2.7 points/month (95%CI 2.1 to 3.3, *p* < 0.001) in patients with 2 copies of survival motor neuron 2 gene. Pneumonia was the most frequently reported adverse event, whereas laboratory tests yielded no unusual findings.

**Conclusion:**

Motor ability of children with SMA were improved under risdiplam with a good safety profile. The degree of motor function improvement was related to the course of disease before treatment.

## Introduction

Among the three drugs that have received approval for the treatment of spinal muscular atrophy (SMA), risdiplam is the latest disease-modifying drug. As a small-molecule survival motor neuron 2 (SMN2) splicing modulator, it increases the retention of SMN2 exon 7, thereby improving the expression of functional SMN protein [1]. The efficacy and safety of risdiplam in SMA types 1–3 have been confirmed in clinical trials such as FIREFISH and SUNFISH [2–5], but real-world data are still lacking. Previous studies have explored the experience of risdiplam therapy in adults with SMA abroad [6–8], while that in Asia population has not been well reported, especially in Chinese SMA children [9–11]. The purpose of this study was to supplement the real-world data by observing the efficacy and safety in SMA children receiving risdiplam in multiple centers in China.

## Methods

### Study design

We retrospectively collected information about children with SMA receiving risdiplam from September 2021 to November 2024 from eight centers in China. All children were diagnosed after symptom onset as 5q SMA with homozygous deletions of exon 7 in the SMN1 gene or compound heterozygous mutations by genetic testing. The study received approval from the Ethics Committee of each participating center. Additionally, informed consent was secured from the children and their families.

### Clinical data collection

Basic information including gender, age of onset, age of initial treatment, SMA subtypes, genetic variants, SMN1 and SMN2 copy number, presence of scoliosis and use of invasive ventilation were extracted from the hospital’s electronic medical record system. Longitudinal data were collected at baseline and during follow-up visits, encompassing treatment duration, number and interval of follow-up visits, scores of motor function scales and laboratory parameters such as blood counts, urinary analysis, liver and kidney functions, as well as adverse events. For patients with SMA type 1, data on event-free survival, which is the proportion of infants surviving without permanent ventilation, were also recorded.

We classified children as type 1, 2 and 3 based on the age of symptom onset and the highest motor milestone achieved. The interval between symptom onset and initiation of treatment was defined as the disease duration before treatment. Based on the mean value of this duration, patients were further categorized into two groups: the short disease duration group and the long disease duration group.

### Motor function scales

The main motor function scales included Children’s Hospital of Philadelphia infant test of neuromuscular disorders (CHOPINTEND), Hammersmith functional motor scale expanded (HFMSE), and revised upper limb module (RULM) [12–14]. Children younger than 2 years old or over the age of 2 who could not sit without support, mainly type 1 and type 2 children, were evaluated by CHOPINTEND scale. Children older than 2 years who could sit without support, mainly type 2 and 3 children, were evaluated by HFMSE or RULM scale [12–14]. Child whose CHOPINTEND scores over 60 is additionally performed by HFMSE. All evaluations were performed under professional trained physicians and clinically significant motor function improvement was defined as an increase of 4.0 or more points on the CHOPINTEND scale, 3.0 or more points on the HFMSE scale, and 2.0 or more points on the RULM scale [12–14].

### Statistical analysis

Categorical data were described by the number of cases, continuous data with normal distribution were reported as mean ± standard deviation, and non-normally distributed measurement data were expressed as median (range). Wilcoxon signed rank test was applied in comparing the motor function scores before and after treatment. Additionally, through generalized estimating equation (GEE), we assessed the association between treatment time and motor function scores in different course of disease before treatment, age of initial treatment, disease types and SMN2 copy number, as well as quantifying monthly improvements in motor ability. *P* < 0.05 (two-sided test) was considered statistically significant. SPSS 25.0 software was utilized for data analyzing.

## Results

### Patients baseline information

In total, 34 SMA patients were collected, comprising 16 males and 18 females, consisting of 15 with SMA type 1, 12 with SMA type 2 and 7 with SMA type 3. Two cases had compound heterozygous variants. 15/34 children had 2 copies of SMN2 and 17/34 children had 3 copies. The age of onset was 7.0 (0.6–33.0) months, and the age of initial treatment was 18.0 (1.0-198.0) months. Seven children had different degrees of scoliosis at baseline, and 1 child required invasive respiration. More details can be found in Table 1.

**Table 1:** Baseline and follow-up information of 34 children with SMA under Risdiplam treatment

No	Sex	SMA subtype	SMN1 copy number and point mutation	SMN2 copy number	Age of onset (months)	Age of initial treatment (months)	Age of last follow-up (months)	Scoliosis	Invasive respiration
1	F	3	0	3	18	35	52	No	No
2	F	1b	0	2	1.4	2	11	No	No
3	M	1b	0	2	2	2	7	No	No
4	M	3	0	3	18	28	32	No	No
5	F	1c	1, c.683T > A	2	4	7	14	No	No
6	F	2	0	3	13	17	20	No	No
7	M	3	0	3	30	60	70	No	No
8	F	1b	0	2	2.7	3	10	No	No
9	M	2	0	3	24	198	202	Yes, 45°	Yes
10	F	2	0	3	24	119	125	No	No
11	M	3	0	4	18	64	87	Yes, 9°	No
12	M	2	0	2	24	44	59	No	No
13	M	2	0	3	15	46	61	No	No
14	F	2	0	3	21	29	38	No	No
15	M	3	0	4	33	37	53	No	No
16	F	2	0	2	7	53	61	Yes, 56°	No
17	F	1b	0	2	1	1	9	No	No
18	M	2	0	3	12	143	148	Yes, 80°	No
19	M	2	0	3	13	18	30	No	No
20	F	1c	1, c.683T > A	2	6	11	14	No	No
21	F	1b	0	2	0.7	1	3	No	No
22	M	1b	0	2	1	3	8	No	No
23	F	1c	0	3	3	3	22	No	No
24	F	1b	0	2	3	7	18	No	No
25	M	2	0	3	9	18	30	No	No
26	M	1c	0	3	3	23	37	No	No
27	M	1b	0	2	1	2	13	No	No
28	F	1b	0	2	2	6	12	No	No
29	F	3	0	3	13	193	196	Yes, 69 °	No
30	M	2	0	3	7	163	172	Yes, 106°	No
31	F	3	0	3	18	182	189	Yes, 73°	No
32	F	2	0	3	18	53	56	No	No
33	M	1b	0	2	0.6	1	6	No	No
34	F	1b	0	2	1	1.5	3.3	No	No

Note: SMA = Spinal muscular atrophy. SMN1 = Survival motor neuron 1. SMN2 = Survival motor neuron 2

### Follow-up information

The follow-up intervals and frequencies varied among the 34 children with different SMA types after treatment. The mean treatment duration was 8.3 ± 4.6 months, during which a total of 55 follow-up visits were conducted. The median interval between follow-up visits was 4.6 months (range: 0.9 to 16.7 months), with the most common interval being 4–5 months (12 out of 55 visits, 21.8%). Children with SMA type 1b were more frequently followed up at intervals of 2–4 months, while those with type 2 and 3 were most commonly followed up at 4-month intervals (Figure S1).

## Outcomes

### Survival and feeding

All infants with type 1 SMA were alive without permanent ventilation or other ventilator support, and none required nasogastric intubation.

### Scores of motor function scales

**Fig. 1:**
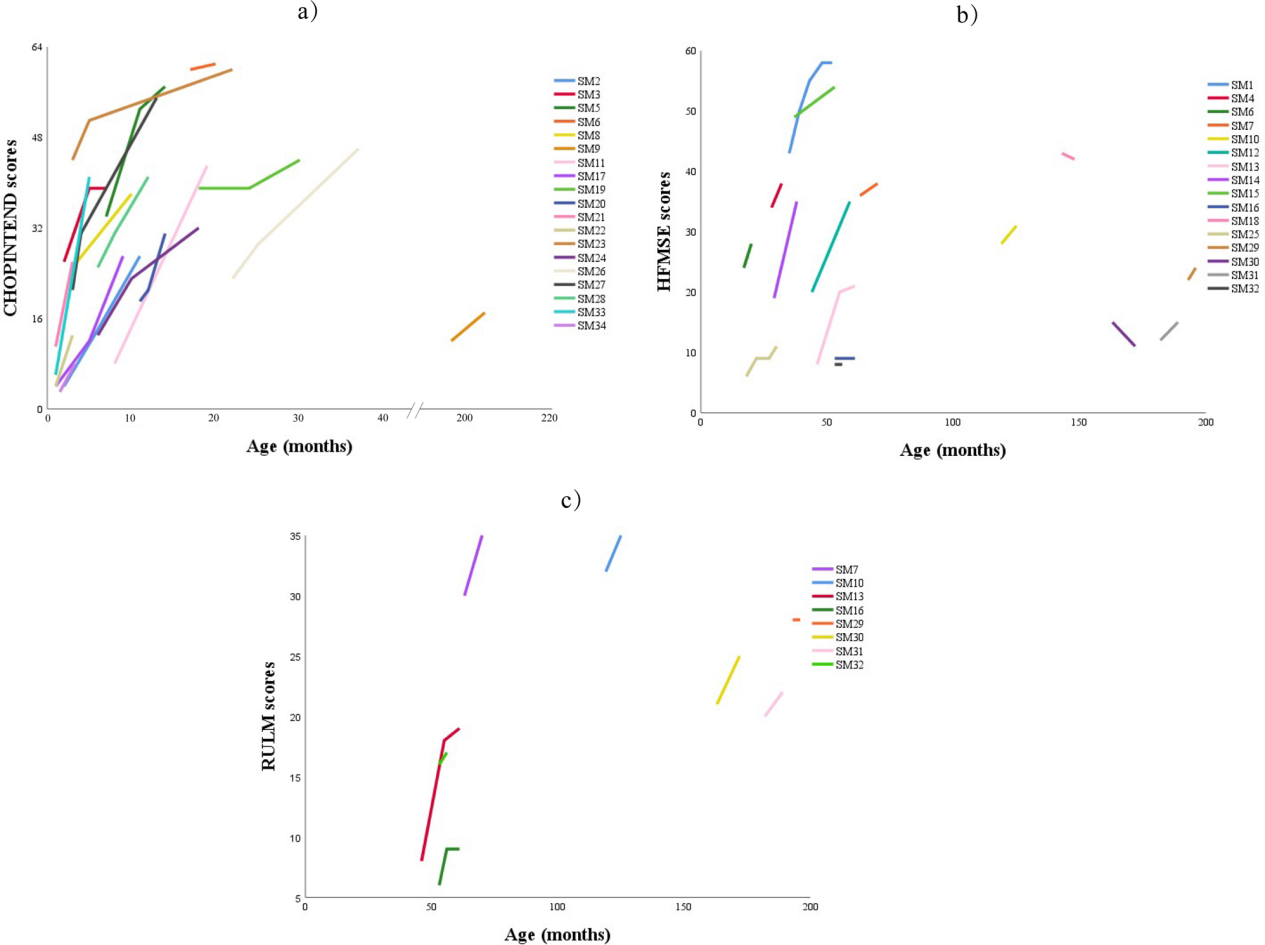
Trends of motor scores in 34 children with SMA under risdiplam treatment in follow-up. **a**) CHOPINTEND = Children’s Hospital of Philadelphia infant test of neuromuscular disorders; **b**)HFMSE = Hammersmith functional motor scale expanded; **c**) RULM = Revised upper limb module. SMA = Spinal muscular atrophy

In general, the scores of motor function scales in most children improved or remained stable, and more details can be found in Fig. 1.

**Fig. 2:**
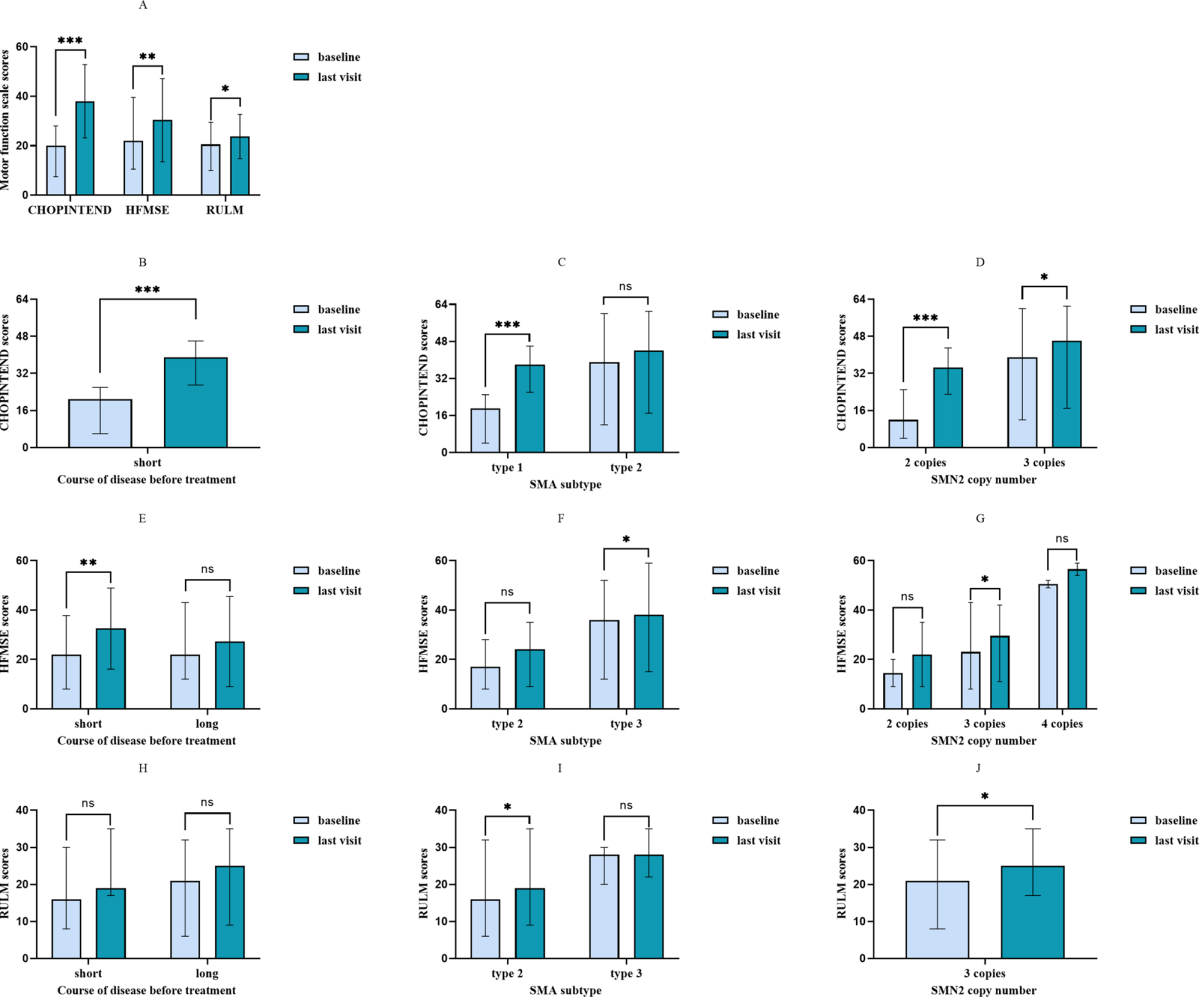
Motor scores at baseline and last time follow-up of 34 children with SMA under risdiplam treatment. Data were expressed as median (range) and statistically analyzed by Wilcoxon signed rank test. **A** showed motor function scores of the analysis in all patients. **B to J** showed motor function scores of the analysis by the course of disease before treatment, SMA subtype and SMN2 copy numbers. CHOPINTEND = Children’s Hospital of Philadelphia infant test of neuromuscular disorders; HFMSE = Hammersmith functional motor scale expanded; RULM = Revised upper limb module; SMA = Spinal muscular atrophy. * *P*<0.05. **P<0.01. ***P<0.001. ns: no significance

Specifically, all 18 children evaluated by CHOPINTEND scale showed an increase in scores at their last visit compared to baseline [20 (3–60) vs. 39 (8–61), *p* < 0.001], and 17(94%) of them experienced clinically significant improvement. Subgroup analysis revealed that the children with shorter disease duration before treatment gained a significant improvement at last visit [21 (3–60) vs. 39 (8–61), *p* < 0.001]. Furthermore, children with type 1 showed statistically significant improvement [19 (3–44) vs. 38 (8–60), *p* = 0.001]. Moreover, improvements were observed when stratifying by SMN2 copy number: children with two copies improved from 13 (3–34) to 31 (8–57) (*p* = 0.001); and those with three copies improved from 39 (12–60) to 46 (17–61) (*p* = 0.042). (Fig. 2A to D).

Seventeen children were evaluated with the HFMSE scale, with 13 children (76.5%) showing improvement at the last follow-up compared to baseline [22 (6–52) vs. 31 (8–59), *p* = 0.003]. Among them, 11 children (64.7%) had clinically significant improvement, while 2 children (11.8%) remained stable. Statistically significant improvements were observed in children with a shorter disease duration before treatment [22 (6–49) vs. 35 (8–58), *p* = 0.008], in those with SMA type 3 [36 (12–52) vs. 38 (15–59), *p* = 0.018], and in those with three copies of the SMN2 gene [22 (6–43) vs. 28 (8–58), *p* = 0.015] (Fig. 2A, E to G).

Seven of eight patients evaluated using the RULM scale showed improvements at the last follow-up compared to baseline [21 (6–32) vs. 24 (9–35), *p* = 0.018], with 6 patients (75%) achieving clinically significant improvement and one patient (12.5%) remaining stable. Patients with type 2 and children with 3 copies number of SMN2 were significantly improved compared with the baseline scores [16 (6–32) vs. 19 (9–35), *p* = 0.042; 21 (8–32) vs. 25 (17–35), *p* = 0.028] (Fig. 2A, H to J).

### Generalized estimating equation model

An analysis of the association between treatment duration and motor scores, using generalized estimating equations, revealed that motor function improvement was positively correlated with treatment duration across various subgroups (Table 2).

**Table 2:** Generalized estimating equation analysis of changes in motor function scales

Outcome	Time			
	B	95%Wald CI		P
		Low	High	
CHOPINTEND				
For all	1.9	0.9	3.0	0.001
Course of disease before treatment				
short course	1.8	0.8	2.9	0.001
SMA subtype				
SMA type 1	2.7	2.4	3.0	<0.001
SMA type 2	0.8	0.5	1.1	<0.001
SMN2 copy number				
2 copies number	2.7	2.1	3.3	<0.001
3 copies number	0.5	0.1	0.9	0.013
HFMSE				
For all	0.2	-0.1	0.5	0.115
Course of disease before treatment				
short course	0.8	0.6	1.0	<0.001
long course	0.0	-0.1	0.1	0.741
SMA subtype				
SMA type 2	0.3	0.1	0.5	0.002
SMA type 3	0.3	0.1	0.5	0.002
SMN2 copy number				
2 copies number	0.9	0.3	1.5	0.005
3 copies number	0.7	0.4	0.9	<0.001
4 copies number	0.2	0.1	0.4	<0.001
RULM				
For all	0.7	0.5	0.9	<0.001
Course of disease before treatment				
short course	0.7	0.7	0.7	<0.001
long course	0.3	0.1	0.4	<0.001
SMA subtype				
SMA type 2	0.7	0.5	0.9	<0.001
SMA type 3	0.1	-0.1	0.4	0.273
SMN2 copy number				
3 copies number	0.7	0.6	0.8	<0.001

Note: CHOPINTEND = Children′s Hospital of Philadelphia infant test of neuromuscular disorders; HFMSE = Hammersmith functional motor scale expanded; RULM = Revised upper limb module; SMA = Spinal muscular atrophy; SMN2 = Survival motor neuron 2; CI = Confidence interval

In terms of disease duration prior to treatment, children in the short-duration group showed a monthly increase of 1.8 points in CHOPINTEND scores (95%CI 0.8 to 2.9, *p* = 0.001), 0.8 points in HFMSE scores (95%CI 0.6 to 1.0, *p* < 0.001) and 0.7 points (95%CI 0.7 to 0.7, *p* < 0.001) in RULM scores. In comparison, children in the long-duration group had a modest improvement in RULM scores with an increase of 0.3 points/month (95%CI 0.1 to 0.4, *p* < 0.001).

Specially, for children with type 1, the CHOPINTEND scores increased by 2.7 points (95%CI 2.4 to 3.0, *p* < 0.001) for every month of treatment. For type 2 children, the CHOPINTEND score increased by 0.8 points/month (95%CI 0.5 to 1.1, *p* < 0.001), HFMSE score increased by 0.3 points/month (95%CI 0.1 to 0.5, *P* = 0.002), and RULM score increased by 0.7 points/month (95%CI 0.5 to 0.9, *p* < 0.001). The HFMSE score of children with type 3 increased by 0.3 points/month (95%CI 0.1 to 0.5, *p* = 0.002).

Regarding SMN2 copy number, children with 2 copies number of SMN2 had an increase of 2.7 points/month in CHOPINTEND (95%CI 2.1 to 3.3, *p* < 0.001) and an increase of 0.9 points/month in HFMSE (95%CI 0.3 to 1.5, *p* = 0.005). Children with 3 copies number of SMN2 showed an increase of 0.7 points/month in RULM (95%CI 0.6 to 0.8, *p* < 0.001).

We additionally analyzed the correlation of motor function with age of initial treatment (Table S1). In type 1 children, the CHOPINTEND score was negatively correlated with the age of initial treatment (*p* < 0.001). The same correlation could be found between HFMSE score (*p* = 0.048) and RULM score (*p* < 0.001) and age of initial treatment in type 2 and 3 children.

### Safety profile

Nine common adverse events were reported, including pneumonia (8.9%), upper respiratory tract infection (5.9%), fever (5.9%), vomiting (3.0%), and orbital pain (3.0%). All adverse events were resolved, and none of these were drug-related according to professional physicians (Table 3). The results of blood parameters were all within the normal range.

**Table 3:** Adverse events during treatment in 34 children with SMA under Risdiplam treatment

Adverse events	Number	Percents
Pneumonia	3	8.9%
Upper respiratory tract infection	2	5.9%
Fever	2	5.9%
Vomiting	1	3.0%
Orbital pain	1	3.0%

Note: SMA = Spinal muscular atrophy

## Discussion

In this study, the motor function of 34 SMA children from multiple centers improved to varying degrees after receiving risdiplam. Notably, children with SMA type 1 demonstrated substantial benefits, while improvements in upper limb function were also observed in those with type 2 and 3, both of which showed a positive correlation with treatment duration. The degree of improvement varied according to SMN2 copy number and was influenced by the disease duration prior to treatment initiation.

The mortality rate of children with type 1 SMA is extremely high in the first 2 years of life [15]. The proportion of patients at 6 months of age who survived without permanent ventilation under natural history was 100%, but decreased to 35% at 12 months age and 10% after 18 months age [16], while in the FIREFISH study, the value at 12 months and 24 months after risdiplam treatment was 85% [2, 4]. In our study, all but 3 patients with type 1 disease were older than 6 months at the last follow-up, have received risdiplam treatment for up to 19 months. All infants are alive and not receiving permanent ventilation, especially type 1b, suggesting that the Chinese children have a good response to risdiplam, demonstrating the efficacy of risdiplam in improving event-free survival. Clinical trials reported that risdiplam significantly improves motor function in infants with SMA [2, 4]. In the FIREFISH study, 90% of infants aged 1 to 7 months with SMA type 1 carrying two copies of SMN2 received improvements of more than 4 scores in CHOPINTEND after 12 and 24 months of treatment [2, 4]. Continuous improvement of 4 points or more in CHOPINTEND scores was also observed for all children in the same population in our study. Patients with type 1c SMA carrying three SMN2 copies also showed improved motor function. Interestingly, three of them who started treatment after 7 months experienced clinically meaningful improvement at the last follow-up, which may supplement the result of previous study but more data are needed to indicate that those with a long history of disease may still benefit from risdiplam treatment.

Our study supplements the real-word data of type 2 and 3 SMA patients in Asian population [11]. HFMSE scale was used to evaluate the overall motor ability of children. A 4-year natural history follow-up of children with type 2 and 3 SMA showed a downward trend in HFMSE scores, with the most dramatic decreases seen in children with type 2 SMA aged 5–14 years and in those with type 3 SMA aged 7–15 years [17]. In our study, 64.7% of children with SMA type 2 and 3 achieved significant improvement, compared with only 26% of children with natural history achieving an increase of ≥ 3 points in the HFMSE score after 15 months [14]. It should be noted that HFMSE showed a decrease trend in 2 children in this study, both had symptom onset before 1 year of age, experienced a longer disease course prior to treatment, and were likely in a period of rapid motor decline based on the natural history of SMA at treatment initiation. Children with SMA have continuous degeneration and loss of motor neurons from birth [18]. We believe that earlier symptom onset and longer disease duration means more motor neuron degeneration and more severe disease progression, which may impact treatment outcomes. However, one of this 2 patients exhibited an increase in the RULM score, which probably suggest that RULM scale is more suitable to evaluate the improvement of upper limb function in children. Previous researchers followed up adult patients, observing that risdiplam demonstrated a great improvement in upper limb function [8] and RULM scale showed greater responsiveness in detecting changes [6]. Higher percentages of children with an increase of ≥ 2 points in RULM scores at our group than in natural history (75% vs. 31%) [3].

We innovatively quantified variations in motor function based on disease duration prior to treatment, SMA types and SMN2 copy numbers. The CHOPINTEND score showed the greatest monthly improvement in children with type 1 and with 2 copies of SMN2. Children with shorter disease duration before treatment had an increase of 1.8 points/month in CHOPINTEND score. The CHOP-INTEND scores showed noticeable improvement within 2 to 3 months, suggesting that even within a short monitoring period, children with earlier initiation of treatment can exhibit clinically meaningful improvements, indicating greater potential benefit. In contrast, in patients with type 2 and 3, HFMSE scores increased by less than 1 point per month, indicating that it may take 4–5 months to observed a meaningful improvement, as well as in RULM scores. Additionally, we observed children with SMA type 1 with 3 copies number of SMN2 when compared to FIREFISH study [19] with an observable increase in CHOPINTEND scores but an insignificant difference in a statistical subgroup analysis. We speculated that due to the short follow-up period, it was not sufficient to observe a statistically significant improvement in scores. The motor function score was negatively correlated with the age of initial treatment, in line with real-world results for other treatments [20], which meant that the earlier the treatment was performed, the better the outcome was.

Common adverse events reported in the previous literature include upper respiratory tract infection, pneumonia, fever, etc [2–5, 21]. In our study, pneumonia was the most common adverse event, with no adverse events deemed related to drug, implying a good safety profile of risdiplam.

In the real world, many factors affect the follow-up interval of children, for example, improvement of drug accessibility, the good efficacy of drugs, and absence of adverse events, which can give patients less incentive for routine follow-up. While the follow-up intervals of these children varied widely, the follow-up interval of children with different types primarily clustered around the 4–5 months as physician may reference nusinersen’s treatment plan which requires hospital visits once every 4 months. Currently, there is no established protocol for the follow-up intervals in patients treated with risdiplam. In the clinical trial, children with type 1 were followed every 2 months in the FIREFISH study and children with type 2 and 3 were followed every 4 months in the SUNFISH study [2, 3]. Kwon et al. recommended children with type 1 or 2 follow up every 3 or 6 months based on age and weight for safety reasons [22], while Hahn et al. suggested two visits per month to facilitate adjustment of drug doses [23]. In Iran multicenter study of the efficacy of risdiplam and nusinersen, children with type 2 and 3 were followed at 3-month intervals for up to 6 months [24]. In this study, the child with type 1b revisited at an interval of about 2 months after initial treatment due to safety concerns. During the follow-up treatment, the patients were followed up at an interval of nearly 4 months, and no adverse events were found. Children with type 1 SMA experienced between 4.2 and 7.6 hospitalizations every year under natural history while the number decreased to 0.63 per year over 48 months of treatment [25]. In our study population, children with type 1b had a maximum interval of 16.7 months to visit the hospital for follow-up, which indirectly implied a long period without adverse events. Despite the favorable safety profile of long-term use of risdiplam, this interval between assessments is not recommended. In conclusion, factors such as changes in condition, efficacy evaluations and safety monitoring should be fully considered when establishing follow-up intervals. The result of quantifying monthly improvements in our research could also be taken in consideration. A follow-up interval of 4 months is operable considering both safety and efficacy.

The study was somewhat restricted by its small sample size and heterogeneity of samples, so the conclusions drawn cannot be well extrapolated to the whole disease population and a larger sample and longer follow-up time are needed to draw further results. However, our findings complement the FIREFISH study, especially for children with type 1b. We describe real-world data from children with type 2 and 3 in Asia for the first time, as well as provide actionable insights into follow-up intervals for risdiplam therapy, which can serve as a reference for standardizing follow-up protocols in the future.

## Conclusion

This retrospective study, the first to present data from Asia, showed that 100% event-free survival with risdiplam in children with type 1 SMA, particularly those with type 1b. The improvement of motor function in children with different types and copy numbers was observed and all children got motor function improved related to the course of disease before treatment, with a good safety profile.

## Data Availability

The data supporting the findings of this study are available on request from the corresponding author. The data are not publicly available due to privacy or ethical restrictions.

## Abbreviations

SMA: Spinal muscular atrophy
SMN: Survival motor neuron
CHOPINTEND: Children’ s Hospital of Philadelphia infant test of neuromuscular disorders
HFMSE: Hammersmith functional motor scale expanded
RULM: Revised upper limb module
GEE: Generalized estimating equation
